# *Chlamydia trachomatis* Frequency in a Cohort of HPV-Infected Colombian Women

**DOI:** 10.1371/journal.pone.0147504

**Published:** 2016-01-25

**Authors:** Edith Margarita Quinónez-Calvache, Dora Inés Ríos-Chaparro, Juan David Ramírez, Sara Cecilia Soto-De León, Milena Camargo, Luisa Del Río-Ospina, Ricardo Sánchez, Manuel Elkin Patarroyo, Manuel Alfonso Patarroyo

**Affiliations:** 1 Molecular Biology and Immunology Department, Fundación Instituto de Inmunología de Colombia (FIDIC), Bogotá, Colombia; 2 Biotechnology Institute, Faculty of Sciences, Universidad Nacional de Colombia, Bogotá, Colombia; 3 Grupo de Investigaciones Microbiológicas–UR (GIMUR), Faculty of Natural and Mathematical Sciences, Universidad del Rosario, Bogotá, Colombia; 4 School of Medicine and Health Sciences, Universidad del Rosario, Bogotá, Colombia; 5 School of Medicine, Universidad Nacional de Colombia, Bogotá, Colombia; Penn State University School of Medicine, UNITED STATES

## Abstract

**Background:**

*Chlamydia trachomatis* (*C*. *trachomatis)*, an obligate intracellular bacterium, is the commonest infectious bacterial agent of sexual transmission throughout the world. It has been shown that the presence of this bacteria in the cervix represents a risk regarding HPV persistence and, thereafter, in developing cervical cancer (CC). Prevalence rates may vary from 2% to 17% in asymptomatic females, depending on the population being analysed. This study reports the identification of *C*. *trachomatis* in a cohort of 219 HPV-infected Colombian females.

**Methods:**

*C*. *trachomatis* infection frequency was determined during each of the study’s follow-up visits; it was detected by amplifying the cryptic plasmid sequence by polymerase chain reaction (PCR) using two sets of primers: KL5/KL6 and KL1/KL2.

Infection was defined as a positive PCR result using either set of primers at any time during the study. Cox proportional risk models were used for evaluating the association between the appearance of infection and a group of independent variables.

**Results:**

Base line *C*. *trachomatis* infection frequency was 28% (n = 61). Most females infected by *C*. *trachomatis* were infected by multiple types of HPV (77.42%), greater prevalence occurring in females infected with HPV-16 (19.18%), followed by HPV-58 (17.81%). It was observed that females having had the most sexual partners (HR = 6.44: 1.59–26.05 95%CI) or infection with multiple types of HPV (HR = 2.85: 1.22–6.63 95%CI) had the greatest risk of developing *C*. *trachomatis*.

**Conclusions:**

The study provides data regarding the epidemiology of *C*. *trachomatis* /HPV coinfection in different population groups of Colombian females and contributes towards understanding the natural history of *C*. *trachomatis* infection.

## Introduction

*Chlamydia trachomatis* (*C*. *trachomatis*) is an obligate intracellular bacterium which can infect both ocular and genital tissues. Infection by *C*. *trachomatis* causes chronic inflammation damaged epithelial tissue and pelvic inflammatory disease (PID). Due to being obligate and intracellular, the pathogen persists in a host after an immune response has been developed, meaning that it could produce chronic disease, causing damage to tissue, resistance to apoptosis and affecting host ability to clear other STI infections [1, 2].

*C*. *trachomatis* is the commonest infectious bacterial agent of sexual transmission throughout the world [3, 4]. The WHO has estimated that about 98 million adults were infected by *C*. *trachomatis* in 2005 and 101 million new cases of *C*. *trachomatis* occur annually around the world [4, 5]. Prevalence rates may vary from 2% to 17% in asymptomatic females, depending on the population and country evaluated [6]. *C*. *trachomatis* prevalence has been reported as being 1.4% for males and 1.6% for females in people aged 18–44 years old, the highest rates occurring in subjects aged 18 to 29 years old: 2.5% for males and 3.2% for females. Its prevalence in sexually-transmitted infection (STI) detection centres has been seen to rise to 15% [7].

*C*. *trachomatis* infection prevalence and determinants in Colombia have not been described completely. Some data have been reported by the Colombian Ministry of Health and Social Protection indicating that 1,538, 1,525 and 1,313 people were diagnosed as being infected by *C*. *trachomatis* in 2009, 2010 and 2011, respectively. It has also been indicated that this is the most prevalent STI in people aged 15 to 49 years-old [8].

The asymptomatic nature of *C*. *trachomatis* infection, its long-term persistence and its ability to induce chronic inflammation and metaplasia has led to this bacteria being considered a potential cofactor in high-risk (HR) human papilloma virus (HPV) infection since it could promote the virus’ persistence and/or potentiate its oncogenicity [2]. Besides inducing tissue inflammation, it also affects host ability to purge HPV infection, thereby contributing towards viral persistence and increasing the risk of developing lesions having a poor prognosis [9].

HR-HPV types are considered the main aetiological agents of cervical neoplasia (CN) [10], however, only a small percentage of HPV-infected females progress to invasive cervical cancer (ICC), meaning that developing it has been associated with other cofactors acting together with HPV [11], such as host-related (e.g. endogenous hormones, genetic background and immune response) and virus-related ones (e.g. load, viral integration and concomitant infection by other STI agents such as HIV and *C*. *trachomatis*) [12].

The importance of such infection in terms of public health means that HPV and *C*. *trachomatis* coinfection frequency must be estimated regarding females with/without cervical lesions [10] associated with possible reinfection and final outcome, bearing in mind that identifying *C*. *trachomatis* when there is coinfection with HPV has been poorly evaluated in Colombia to date [13]. This study was thus aimed at determining *C*. *trachomatis* frequency in a population of HPV-infected females living in 3 cities in Colombia (Bogotá, Chaparral and Girardot). This is the first study which has evaluated a cohort which was completely infected with HPV at the start; the aim was to determine *C*. *trachomatis* infection incidence and the risk factors associated with HPV coinfection.

## Materials and Methods

### Study population

The population studied consisted of 219 females whose ages ranged from 17–71 years old. A previous study has described how the cohort was assembled for identifying HPV, involving 3 follow-ups every 6 months (± 3 months) [14].

The females in the study were living in 3 cities in Colombia: Bogotá, the capital city, Chaparral in the Tolima department and Girardot in the Cundinamarca department, where intrinsic characteristics have been identified in previous studies regarding the risk of contracting HPV infection [15]. Chaparral and Girardot were grouped under the “other city” category to facilitate statistical analysis.

### Ethics, consent and permissions

The females involved in the study carried out by the Fundación Instituto de Inmunología de Colombia (FIDIC) voluntarily decided to participate in sampling between April 2007 and March 2010; they signed an informed consent form which explained the risk factors associated with the exams and sample taking. A parent or guardian/teacher had to sign for girls aged less than 18 years old. All the procedures were approved by the following hospitals’ ethics’ committees: Hospital de Engativá (Bogotá population), Hospital San Juan Bautista (Chaparral) and Nuevo Hospital San Rafael (Girardot). *C*. *trachomatis* was determined from samples taken from the 219 HPV-infected females at the start of the follow-up and thereafter during each visit whilst the study lasted.

### Obtaining biological material

Biological material was obtained from cervical smear samples collected after cytology and stored at -20°C. The samples were processed and DNA extracted, according to information previously published by our group [14].

### Molecular detection of *C*. *trachomatis*

Polymerase chain reaction (PCR) amplification of a sequence from the microorganism’s cryptic plasmid was used for detecting *C*. *trachomatis* in samples from HPV-infected females. Two sets of primers previously reported in the literature were used; KL5/KL6 [16, 17] amplified a 350 bp conserved cryptic multicopy plasmid fragment and KL1/KL2 a 241 bp fragment [2, 18, 19] (Table 1). The reactions with each set of primers were done separately and simultaneously for each sample.

**Table 1:** Primers used for amplifying *Chlamydia trachomatis*.

Region	Name of primer	Sequence
**pLGV440**	**KL5**	5’- TTT GCC TTA ACC CCA CCA TT-3’
	**KL6**	5’- CGT CCT TCC TAA AAG AGC TA -3’
**pLGV440**	**KL1**	5’- TCC GGA GCG AGT TAC TAA GA -3’
	**KL2**	5’- AAT CAA TGC CCG GGA TTG GT -3’

Amplification conditions were chosen and adjusted according to the methodologies described for *C*. *trachomatis* detection [2, 10, 18, 19]. The *C*. *trachomatis* ATCC UW-36/Cx strain (ATCC VR-886D) was used as positive control for each amplification and DNAse-free water as reagent control. The PCR for each set of primers was done at 20 μL final volume, using 3 μL of sample, with approximately 900 ng of DNA.

Reaction conditions for KL5 and KL6 were: 1X concentration Bioline buffer (KCl, Tris HCl, pH = 8.3), 3mM MgCl_2_, 1.25mM each desoxinucleotide triphosphate (dNTP), 20 pmol of each primer and 1.25 μL of Taq DNA polymerase (5U/μL). This set of primers’ thermal profile involved an initial cycle at 94°C for 5 minutes, followed by 35 cycles of 1 minute at 93°C, 1 min at 56.8°C, 1 min at 72°C and a final extension step at 72°C for 10 minutes.

Reaction conditions for KL1 and KL2 were: Bioline buffer (KCl, Tris HCl pH = 8.3) at 1X concentration, 3mM MgCl_2_, 1.25mM each desoxinucleotide triphosphate (dNTP), 20 pmol of each primer and 1.25 μL of Taq DNA polymerase (5U/μL) (Bioline). This set of primers’ thermal profile involved an initial cycle at 94°C for 5 minutes, followed by 35 cycles of 1 minute at 93°C, 1 min at 55°C, 1 min at 72°C and a final extension step at 72°C for 10 minutes.

Bovine serum albumin (BSA) was added to the molecular biology grade water used in the PCR reactions at 0.8 μg/μL concentration; this was added to increase PCR efficiency as described earlier [20].

### Statistical analysis

*C*. *trachomatis* infection frequency was determined during each follow-up visit of the study. Means and their corresponding measures of dispersion (standard deviation) were used for describing continuous variables. Categorical variables were expressed in terms of frequencies and percentages.

The association between the presence of infection and a group of categorical variables was evaluated; these were: smoking, city, ethnicity, age on first sexual relationship, number of sexual partners, family planning method, presence of sexually-transmitted infection (STI), number of children, history of abortions, coinfection with HR-HPV, HPV16, HPV18, HPV31, HPV45, HPV33, HPV58, the amount of HPV infecting types, cytology (negative, ASC-H, ASC-US, LIE-BG, LIE-AG), colposcopy (negative, LIE-BG, LIE-AG, suggestive, unsatisfactory), using Chi^2^ (χ^2^) and Fisher’s exact tests, depending on contingency table characteristics.

*C*. *trachomatis infection* was defined as a positive PCR result using either set of primers at any time during the study. Clearance was defined as *C*. *trachomatis* not being detected by both sets of primers following a positive finding during the previous visit. Persistence was defined as the presence of infection in two consecutive visits. Coinfection was defined as the simultaneous presence of *C*. *trachomatis* and HPV infection whilst the coexistence of two or more types of HPV was defined as multiple infection; such definitions were used according to that established in previous studies [21–23].

Two subpopulations were defined for the cohort study; the first involved patients who were not infected by *C*. *trachomatis* when the cohort study began (to evaluate infection incidence) whilst the second subpopulation involved females who were infected from the start (for evaluating clearance). Follow-up times were used for estimating Kaplan-Meier survival functions for each outcome (infection or clearance).

Infection (for those who started without *C*. *trachomatis*) and clearance rates (for those who were infected at the beginning) were estimated according to the information obtained. Time was calculated in terms of months elapsed since the beginning of follow-up until the event in question (infection or clearance). Cases where no event occurred and follow-up had ended were handled as right censoring for survival analysis.

Cox proportional risk models were used for evaluating the association between the appearance of infection and a group of independent variables, such as race, smoking, age on first sexual relationship, family planning method, number of sexual partners, age, number of children, the presence of sexually-transmitted infection (STI) and coinfection with HR-HPV. The beginning of risk was assumed to be when the cohort began without infection and with follow-up until the presence of an infection event, or the end of the study considered a closed case. The strength of association between the independent variables and the risk of having infection was quantified by using a hazard ratio (HR) estimator, i.e. the ratio of hazard rates for the independent variables’ different levels.

The hypothesis tests involved using 5% significance (p ≤ 0.05), 95% confidence interval (CI) for the estimator and two-tail hypothesis. STATA 12 software was used for all statistical analysis.

## Results

### The cohort’s characteristics

Two hundred and nineteen cervical samples from females who stared the cohort infected by HR-HPV (HPV-16, -18, -31, -33, -45 and -58) were analysed; these females were followed-up every 6 (±3) months. All the females made a minimum of 3 visits (n = 216); however, some of them (n = 49) made 5 visits. A positive or negative *C*. *trachomatis* infection result during visit 0 (base line visit) was taken as starting point for further analysis; *C*. *trachomatis* was positively identified in 57 females during visit 0, whilst 157 did not have *C*. *trachomatis* during the same visit.

**Fig 1:**
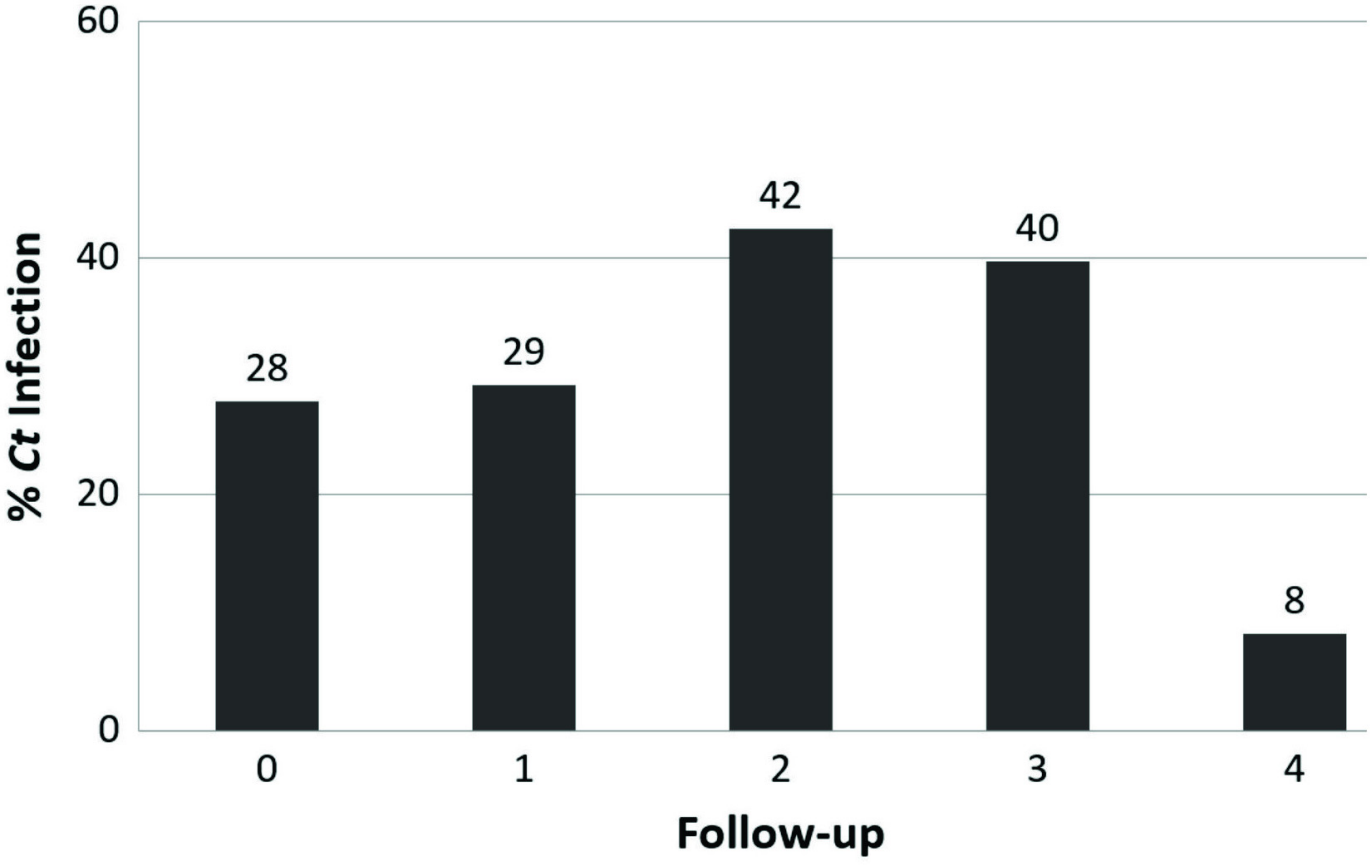
Percentage of females infected by *C*. *trachomatis* per visit.

Sixty-eight of the 219 females came from the Bogota population and 151 belonged to the category “other city”; 40.32% (n = 25) of the females infected by *C*. *trachomatis* at the start of the cohort lived in Bogotá and the remaining 59.68% (n = 37) in a population classed as “different to Bogota”. Table 2 describes the sociodemographic and clinical variables and risk factors from the information supplied at the start of follow-up, according to *C*. *trachomatis* infection state. The highest detection rate was obtained during follow-up visit 2 where the highest level of *C*. *trachomatis* infection was observed (Fig 1).

**Table 2:** The distribution of socio-demographic characteristics and risk factors.

	Chlamydia identification				
	Negative		Positive		P
	n	%	n	%	
**Smoker**					
**No**	117	84.78	50	89.29	0.42
**Yes**	21	15.22	6	10.71	
**City**					
**Bogota**	43	27.39	25	40.32	0.062
**Other**	114	72.61	37	59.68	
**Ethnicity**					
**White**	3	1.91	0	0	0.606
**Indigenous**	1	0.64	1	1.61	
**Mestizo**	151	96.18	61	98.39	
**Black**	2	1.27	0	0	
**Age on first sexual relationship**					
**< = 18**	112	71.34	38	61.29	0.149
**>18**	45	28.66	24	38.71	
**No of sexual partners**					
**1**	77	49.04	25	40.98	0.057
**2–3**	56	35.67	18	29.51	
**>3**	24	15.29	18	29.51	
**Family planning method**					
**No method**	69	43.95	28	45.90	0.957
**Hormonal**	17	10.83	6	9.84	
**Other**	71	45.22	27	44.26	
**STI**					
**HIV**	1	0.65	0	0	0.789
**None**	124	80.52	48	78.69	
**Other**	29	18.83	13	21.31	
**No of children**					
**0**	8	5.10	3	4.92	0.495
**1–2**	82	52.23	36	59.02	
**3–4**	59	37.58	17	27.87	
**>4**	8	5.10	5	8.20	
**No of abortions**					
**No**	93	62	38	64.41	0.746
**Yes**	57	38	21	35.29	
**Multiple HPV infections**					
**No**	38	24.20	14	22.58	0.799
**Yes**	119	75.80	48	77.42	
**HPV16**					
**No**	44	28.03	20	32.26	0.535
**Yes**	113	71.97	42	67.74	
**HPV18**					
**No**	68	43.31	35	56.45	0.079
**Yes**	89	56.69	27	43.55	
**HPV31**					
**No**	103	66.88	32	51.61	0.079
**Yes**	51	33.12	30	48.39	
**HPV45**					
**No**	76	48.41	48	77.42	0.000
**Yes**	81	51.59	14	22.58	
**HPV33**					
**No**	145	92.36	60	96.77	0.359
**Yes**	12	7.64	2	3.23	
**HPV58**					
**No**	96	61.15	23	37.10	0.001
**Yes**	61	38.85	39	62.90	
**Number of HPV infecting types**					
**1**	38	24.20	14	22.58	0.119
**2**	34	21.66	22	35.48	
**3**	54	34.39	12	19.35	
**4**	18	11.46	11	17.74	
**5**	9	5.73	2	3.23	
**6**	4	2.55	1	1.61	
**Cytology** ^a^					
**Negative**	551	94.67	287	93.79	0.76
**ASC-H**	1	0.17	0	0.00	
**ASC-US**	14	2.41	6	1.96	
**L-SIL**	15	2.58	12	3.92	
**H-SIL**	1	0.17	1	0.33	
**Colposcopy**					
**Negative**	477	89.66	259	90.88	0.296
**L-SIL**	53	9.96	23	8.07	
**H-SIL**	1	0.19	2	0.70	
**Suggestive**	0	0.00	1	0.35	
**Unsatisfactory**	1	0.19	0	0.00	

^a^ ASC-H: atypical squamous cells not exclude H-SIL; ASC-US: atypical squamous cells of undetermined significance; L-SIL: low squamous intraepithelial lesion; H-SIL: high squamous intraepithelial lesion.

The percentage of females infected by multiple types of HPV (77.42%) proving positive for *C*. *trachomatis* infection was much greater than that for those having just a single HPV infection (22.58%). Regarding *C*. *trachomatis* coinfection and different types of HR-HPV, it was found that type 16 was present in most females in the cohort (51.59%) compared to the total population, followed by types 18 (40.63%), 45 (36.98%), 58 (27.85%), 31 (23.28%) and 33 (5.47%). The greatest *C*. *trachomatis* frequency rate for multiple HPV infection was found in HR-HPV type 16 (19.18%) followed by type 58 (17.81%). A statistically significant difference was observed between populations infected by *C*. *trachomatis* and those without infection regarding females infected by HR-HPV 58 and 45.

**Fig 2:**
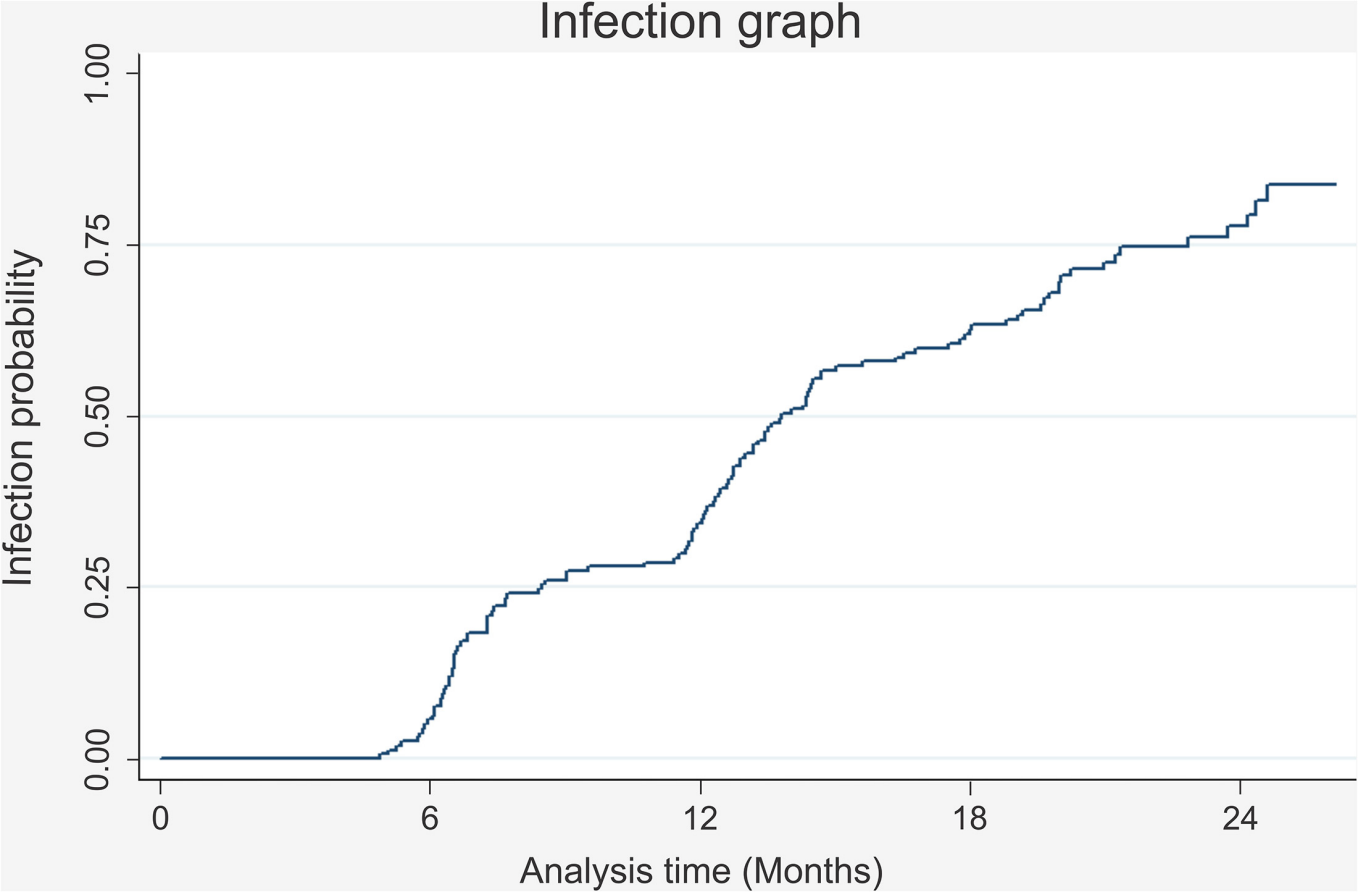
The probability of the risk of the women in this cohort acquiring *C*. *trachomatis* infection as time elapsed.

**Fig 3:**
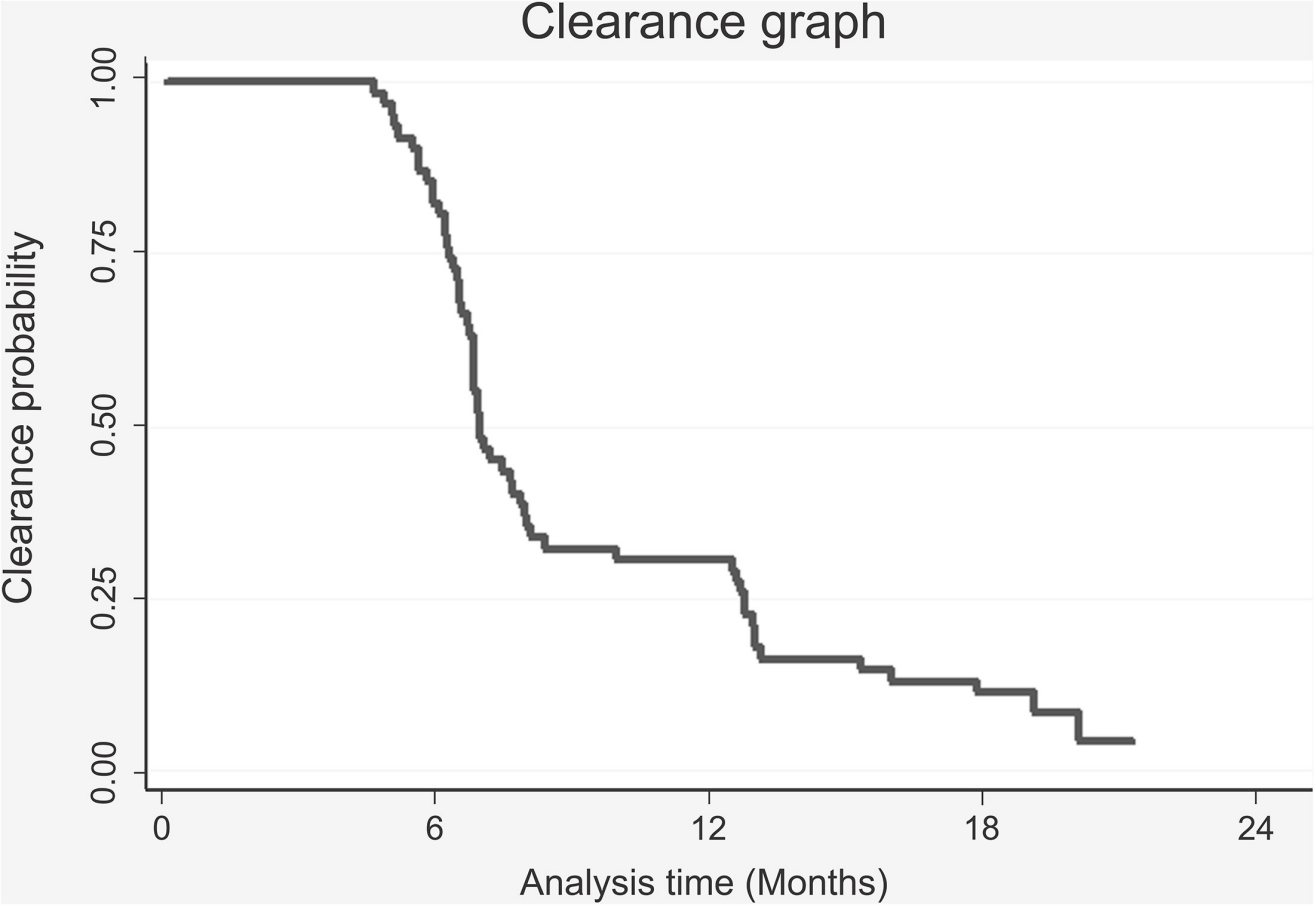
The time taken to clear *Chlamydia trachomatis* infection in the cohort of females initially infected by HPV and *C*. *trachomatis*.

For females who began the study without infection, the total time contributed towards the study cohort was 2,281 months; 74.52% (n = 117) of them became infected, whilst 25.47% (n = 40) remained *C*. *trachomatis* -free during total follow-up time (around 2 years). Infection rate was 5.1 per 100 people during one month (4.27–6.14 95%CI). Fig 2 shows the probability of the risk of acquiring the infection throughout the follow-up period for the group of women in the cohort who began without having *C*. *trachomatis* infection. Fig 3 shows the probability of clearing *C*. *trachomatis* infection in time.

Females who began with infection contributed 587 months at risk to the study cohort, infection rate being 9.7 per 100 people in a month (7.48–12.58 95%CI); 8.06% (n = 5) of the females who started infected did not clear the infection during the time the study lasted.

Base line *C*. *trachomatis* infection frequency was 28% (n = 61). There was 85% (n = 52) *C*. *trachomatis* identification by the KL1/KL2 and 29% (n = 18) with KL5/KL6 set of primers in base line data. The detection limit for each set of primers was calculated, the results showing that KL1/KL2 detected lower amounts of *C*. *trachomatis* DNA (48.2 μg/μL) compared to the KL5/KL6 set of primers (57.6 μg/μL).

Different variables associated with the risk of *C*. *trachomatis* infection were analysed in this study, such as race, smoking, age on first sexual relationship, the family planning method used, the number of sexual partners, age, the number of children, the relationship of *C*. *trachomatis* coinfection with multiple HPV infection and the presence or absence of other STI. Amongst these variables, it was observed that females having had the most sexual partners (HR = 6.44: 1.59–26.05 95%CI) or infection with multiple types of HPV (HR = 2.85: 1.22–6.63 95%CI) had the greatest risk of developing *C*. *trachomatis* infection (Table 3). Moreover, females using a hormonal planning method (HR = 0.33: 0.13–0.79 95%CI) had a reduced risk of developing *C*. *trachomatis* infection compared to those who did not use any conceptive method (Table 3).

**Table 3:** Cox model.

Infection risk factors for			
*Chlamydia trachomatis*	HR (95%CI)^a^		P
**Race**			
**Mestizo**	Reference		0.343
**Other**	1.71	(0.56–5.22)	
**Smoker**			
**No**	Reference		0.110
**Yes**	0.26	(0.04–1.35)	
**Age on first sexual relationship**			
**< = 18**	Reference		0.345
**>18**	1.26	(0.77–2.06)	
**Family planning method**			
**No method**	Reference		
**Hormonal**	**0.33**	**(0.13–0.79)**	0.013
**Other**	0.85	(0.52–1.38)	0.523
**No of sexual partners**			
**1**	Reference		
**2–3**	2.11	(0.63–7.00)	0.221
**>3**	**6.44**	**(1.59–26.05)**	0.009
**Age**			
**< = 35**	Reference		
**36–45**	0.92	(0.50–1.68)	0.804
**>45**	0.52	(0.26–1.04)	0.067
**Children**			
**0**	Reference		
**1–2**	1.29	(0.43–3.85)	0.643
**>3**	1.54	(0.49–4.79)	0.455
**HR-HPV type coinfection**			
= **1**	Reference		0.738
**2–4**	0.90	(0.51–1.60)	
**>4**	**2.85**	**(1.22–6.63)**	0.015
**STI**			
**1**	Reference		0.168
**2**	0.63	(0.33–1.20)	

Values in bold = p < 0.05.

^a^ Hazard ratio (HR), adjusted for smoking, city, ethnicity, age on first sexual relationship, number of sexual partners, family planning method, presence of sexually-transmitted infection (STI), the number of children, abortions, confection with HR-HPV, HPV16, HPV18, HPV31, HPV45, HPV33, HPV58, number of HPV infecting types.

## Discussion

This study involved a group of 219 females whose ages ranged from 17–71 years old. The estimated base line *C*. *trachomatis* frequency rate was 28% in a cohort of HPV-infected females from different populations in Colombia, such percentage being higher than that reported in studies in countries like Italy (17%) [24] and Argentina (16.3%) [2] where the samples examined were coinfected with HPV. The aforementioned 28% was also higher than that reported to date for Colombia (5.0%) [13]; however, it should be stressed that the *C*. *trachomatis* infection rates in the only Colombian study to date were estimated in a general population of women.

Identifying greater prevalence than that reported in other studies relied on two significant factors. This cohort represented a population at risk since all the females were infected by HPV when the cohort started, meaning that this population was probably engaging in behaviour leading to the risk of acquiring an STI [25]. The second factor was the high sensitivity of the detection technique used here (PCR) [26], as well as two different primer sets being used for *C*. *trachomatis* identification, thereby increasing the chance of finding positive samples. Previous studies have reported the individual use of the primer sets tested here in samples from HPV-infected women, showing 13% frequency for KL5/KL6 and 34.9% for KL1/KL2 [2, 27].

Regarding the risk factors associated with acquiring *C*. *trachomatis* infection, an increased number of sexual partners was found, thus agreeing with previous reports describing a similar association not only with *C*. *trachomatis*, but with almost all STI [28–30]. The fact of having had many sexual partners also implies being involved in risky sexual behaviour thereby facilitating contagion by more than one type of microorganism and hampering clearance of infection [3, 10, 31–33].

Another relevant factor found was infection by multiple HR-HPV types, similar to what has been described in other Latin American countries [10, 27, 34]. The increased risk has been biologically explained by studies showing that *C*. *trachomatis* infection favours the entry and persistence of multiple HR-HPV types which leads to viral integration, apoptosis inhibition, overexpression of the E6 / E7 oncogenes and cellular transformation [35]. Bearing in mind *C*. *trachomatis* pathogeny and the tissue damage produced in chronic infections, this epithelium could become affected by various types of HPV virus, leading to multiple infections, as observed in this study. This has been reported in a previous study involving multiple detection of HPV-16 and -18 genotypes [32].

Regarding *C*. *trachomatis* coinfection with different types of HR-HPV, it was found that types like HPV-16 and -18 occurred most frequently, similar to that reported in a population of female aborigines in the north of Argentina [12]; however, it has been found that HPV-18 and -52 occurs most frequently in the Egyptian population [32]. Such data could suggest that genetic factors might modulate this association or depend on the specific type of HPV distribution in a particular region being analysed; however, further studies are needed regarding the HPV-infected population to elucidate such differences.

The most common *C*. *trachomatis* coinfection with HPV-HR in the cohort being studied involved type 16. It has been reported in the literature that *C*. *trachomatis* infection improves Ki67 protein expression, this being a marker for cervical epithelium cell proliferation; the same is true for HPV infection, especially that including HPV-16 [12]. It has been found that *C*. *trachomatis* infection increased HPV-16 expression in CIN I, suggesting that it could modify the activity of this type of virus; it has also been confirmed that *C*. *trachomatis* infection has increased EGFR and TGF-alpha expression and this could explain distinct variants of the cervical carcinogenesis mechanism [36].

It is worth mentioning that HR-HPV 58-infected women had the second highest *C*. *trachomatis* infection frequency in our study which might have been related to a pattern of multiple HPV infection distribution in our population, since a previous report has shown that HPV-58 and -45 are usually present in multiple infections [14].

It could be seen that around a quarter of the HPV-infected females in this cohort had become infected by *C*. *trachomatis* six months after the study began and that half the population had become infected after one year’s follow-up. On the other hand, females who were infected at the start of the cohort required about six months to a year for clearing the infection. This result agreed with a previous study carried out in Bogotá [22] (one of the cities analysed here) which found that 54% of the *C*. *trachomatis* infected women who did not receive therapy resolved the infection in 1 year. No particular variation in infection clearance time was found here when comparing the three geographical populations studied.

Data for asymptomatic *C*. *trachomatis* infected women in Amsterdam had a person/year spontaneous clearance rate of 44.7%. However, the pertinent study stated that genital *C*. *trachomatis* infection duration in the absence of treatment had not been completely elucidated, but available long-term studies suggest that a typical untreated *C*. *trachomatis* infection can last a year or longer before clearance [21, 37].

The present study found that females in the cohort using a hormone-based family planning method had less risk of developing *C*. *trachomatis* infection; this has been supported by studies in animal models concluding that estradiol reduces the susceptibility to intrauterine *C*. *trachomatis* infection in rats [38]. Other authors have found that oral contraceptive use has been associated with a faster *C*. *trachomatis* infection clearance rate [22].

A weakness of this study of a cohort of HPV-infected females was that it lacked parallel screening involving *C*. *trachomatis* in a population of females who were not infected by this virus for statistically correlating frequency in both groups, thereby contributing towards assessing *C*. *trachomatis* infection rates in the Colombian population. Determining and analysing the types of *C*. *trachomatis* in patients having cytological findings would establish whether the relationship of both agents came from mutual potentiation rather than the fact that they share a common transmission route.

## Conclusions

The study provides data regarding the epidemiology of *C*. *trachomatis*/HPV coinfection in different population groups of Colombian females. Having had more sexual partners and infection by multiple types of HPV is associated with a greater risk of becoming infected by *C*. *trachomatis*. Using hormonal contraceptives was a factor for the cohort being studied, as significant importance was found regarding an association with lower risk of becoming infected by *C*. *trachomatis*. This paper contributes towards understanding the natural history of *C*. *trachomatis* infection.

## Abbreviations

*C. trachomatis*: *Chlamydia trachomatis*
HPV: human papillomavirus
HR-HPV: high-risk human papillomavirus
CC: cervical cancer
CIN: cervical intraepithelial neoplasia
PCR: polymerase chain reaction
DNA: deoxyribonucleic acid
STI: sexually-transmitted infection
SD: standard deviation
CI: confidence interval
HR: hazard ratio
ASC-H: atypical squamous cells not exclude H-SIL
ASC-US: atypical squamous cells of undetermined significance
L-SIL: low squamous intraepithelial lesion
H-SIL: high squamous intraepithelial lesion
